# Interactive Effects of Dietary Protein Levels and Magnetic Water Treatment on Water Quality, Growth Metrics, Carcass Composition, Redox Balance, Enzymatic Functions, and Immune Responses in *Oreochromis niloticus*

**DOI:** 10.3390/ani15162388

**Published:** 2025-08-14

**Authors:** Zeinab M. A. Abd-El Azeem, Kareem M. Ahmed, Reham A. Abdelhay, Hossam A. M. Mounes, Adham A. Al-Sagheer, Haytham A. Abd El-Ghaffar, Yasmina M. Abd-Elhakim, Bayan A. Hassan, Dena A. Abd El-Bary

**Affiliations:** 1 Fish Production Branch, Department of Animal Production, Faculty of Agriculture, Ain Shams University, Cairo 11241, Egypt; zeinab_ma83@yahoo.com (Z.M.A.A.-E.A.); kareem_ahmed@agr.asu.edu.eg (K.M.A.); 2 Limnology Department, Central Laboratory for Aquaculture Research, Agricultural Research Center, Abassa, Abu Hammad 44662, Egypt; r_abdelwahab2010@yahoo.com (R.A.A.); hmoans80@hotmail.com (H.A.M.M.); 3 Department of Animal Production, Faculty of Agriculture, Zagazig University, Zagazig 44511, Egypt; 4 Department of Hatchery and Fish Physiology, Central Laboratory for Aquaculture Research, Agricultural Research Center, Abbassa, Abu Hammad 44662, Egypt; dr_haytham1983@hotmail.com; 5 Department of Forensic Medicine and Toxicology, Faculty of Veterinary Medicine, Zagazig University, Zagazig 44519, Egypt; 6 Pharmacology Department, Faculty of Pharmacy, Future University, Cairo 11835, Egypt; 7 Regional Center for Food and Feed, Agricultural Research Center, Giza 12619, Egypt; dinaabbas194@gmail.com

**Keywords:** Nile tilapia, magnetic water treatment, dietary crude protein, growth, antioxidant status, immune response

## Abstract

The present study was conducted to evaluate the individual and combined effects of dietary crude protein levels and magnetic water treatment on the growth performance, water quality, body composition, physiological responses, and immunity of *Oreochromis niloticus*. Using a 3 × 2 factorial design, three levels of dietary crude protein (25%, 30%, and 35%) and two water types (magnetized and non-magnetized) were tested. A total of 180 juvenile tilapia (average initial weight: 4.13 ± 0.004 g) were randomly assigned to six treatment groups and reared for 10 weeks. Results showed that magnetic water treatment significantly improved dissolved oxygen and pH, while reducing ammonia, nitrite, and nitrate concentrations. Growth performance indicators, including final weight, specific growth rate, feed conversion ratio, and average daily gain, were significantly improved by both magnetic water and increased dietary protein. Carcass crude protein content improved with both the higher dietary protein level and magnetic water, while lipid content decreased. Liver and kidney function indicators (AST, ALT, ALP, and urea) were significantly improved by magnetic treatment and higher protein levels. Blood biochemical markers (TP, ALB, and GLO) were elevated, while glucose, cholesterol, and triglycerides were reduced by magnetic water; significant interactions were observed for globulin, triglycerides, and total protein. Antioxidant enzyme activities (SOD, CAT, and GPx) increased, and MDA decreased in response to magnetic water and high-protein diets. Similarly, digestive enzyme activities (lipase, protease, and amylase) and immune parameters (IgM and lysozyme) were significantly improved, with the best values recorded in the 35% protein + magnetic water group. In conclusion, magnetic water treatment and dietary protein level independently and interactively affect the physiological performance and health of Nile tilapia, with the best outcomes obtained at 35% protein under magnetic water conditions.

## Simple Summary

This study presents a novel investigation into the combined effects of magnetic water treatment and dietary protein levels on the growth performance, physiological status, and water quality in Nile tilapia. Magnetic water, produced by exposing water to a magnetic field, has been reported to enhance oxygen availability and reduce nitrogenous waste compounds. Given that high-protein diets in aquaculture can increase the production of ammonia and other nitrogenous metabolites, we evaluated the interaction between magnetic water and three dietary protein levels (25%, 30%, and 35%) over a 10-week feeding trial. The results demonstrated a significant interaction between magnetic water treatment and dietary protein level, with the most pronounced effects observed at the 35% protein level. This combined treatment significantly enhanced the survival rate, reduced urea concentration, and improved serum total protein, globulin, and triglyceride levels, as well as IgM concentration and the activities of lipase and amylase. These findings indicate a synergistic interaction between magnetic water treatment and a 35% dietary protein level, resulting in the most favorable physiological responses in Nile tilapia.

## 1. Introduction

Aquaculture has emerged as a rapidly expanding component of global food production. In 2022, the combined output from aquaculture and fisheries was estimated at nearly 223 million tonnes, reflecting a 4.4% rise compared to 2020 levels [1]. Among the many finfish species cultivated, Nile tilapia (*Oreochromis niloticus*) stands out as one of the most important due to its adaptability to varied farming conditions, disease resistance, and efficient feed utilization [2]. Its broad tolerance to environmental stressors such as poor water quality and high stocking densities further enhances its suitability for intensive production systems [3]. However, optimal performance of tilapia requires well-balanced nutrition and appropriate water quality to support growth, metabolism, and immunity [4].

Dietary protein is the most influential nutrient driving fish growth and health. In fish diets, crude protein levels strongly affect growth rate, feed utilization, immune function, and body composition [5]. In Nile tilapia, optimal protein requirements are typically in the 25–35% range for juveniles, dropping to about 20% for adults [6]. Adequate protein also supports immune and antioxidant defenses, whereas protein deficits can impair growth, muscle development, and disease resistance [5]. Because protein is the costliest feed ingredient, nutritionists strive to optimize levels to balance performance and feed cost [7]. Dietary protein also influences water quality (for example, via ammonia excretion) [8], linking nutrition to the rearing environment.

In parallel to nutrition, maintaining optimal water quality is essential in aquaculture. Magnetic water treatment is an emerging non-chemical approach that aims to improve water by altering its molecular properties [9]. In recent years, the application of electromagnetic fields has expanded across multiple sectors, including wastewater treatment, food processing, agriculture, and aquaculture [10,11,12]. Magnetic exposure has been shown to alter the physicochemical characteristics of water, which in turn influence the biological functions of fish that depend on it [13]. The use of magnetized water has shown promising results in aquaculture, particularly in improving growth and water quality for Nile tilapia. For instance, Hassan et al. [14] reported that a magnetic field of 0.15 tesla (T) significantly enhanced the growth of tilapia, while Ahmed and Abd El-Hamed [9] found that using a magnetic field strength of 0.20 T enhanced growth performance, blood parameters, and water quality in *O. niloticus*. Similarly, Hassan and Rahman [15] found that moderate magnetic fields (0.10–0.20 T) improved hatchability and water quality parameters in Artemia cultures. Furthermore, magnetic field exposure can enhance immune function in aquatic species, as demonstrated by increased immunological markers and improved responses at injury sites [16,17]. These immunological changes are often accompanied by broader physiological benefits, including improved growth, enhanced protein metabolism, and better blood parameters. As highlighted by Rosen [18] and Tang et al. [19], magnetic field technology offers several benefits over traditional chemical methods, including improved organismal performance and reduced mortality in aquaculture settings.

While the individual effects of dietary protein levels and magnetic water treatment on aquaculture species have been previously investigated, their potential interaction and combined influence on fish physiology remain largely unexplored. To our knowledge, no study has examined how varying levels of dietary crude protein may modulate the physiological, immunological, and biochemical responses of fish reared in magnetically treated water. Therefore, the present study aimed to assess the interactive effects of magnetic water treatment and graded dietary protein levels (25%, 30%, and 35%) on growth performance, water chemistry, carcass composition, digestive and antioxidant enzyme activities, and immune indices in *O. niloticus*.

## 2. Materials and Methods

### 2.1. Diets

Three isolipidic (crude lipids: 6.5%) diets were formulated containing 25%, 30%, and 35% crude protein. Before preparing the experimental diets, all ingredients were finely ground to achieve a uniform particle size. The dry ingredients were then thoroughly mixed step by step to ensure homogeneity. Water (approximately 20%) was gradually added to form a well-moistened dough, which was further mixed for even moisture distribution. The mixture was then processed using a pelletizer (California Pellet Mill, San Francisco, CA, USA) to produce 2 mm pellets. These pellets were dried at 45 °C for 8–24 h until the dry matter content reached approximately 90%. Once dried, they were packed in sealed bags and stored at −20 °C to maintain their quality until use. The experimental diets were formulated to fulfill the nutritional requirements of tilapia based on NRC [20] guidelines. The ingredients and nutrient composition of these diets are detailed in Table 1.

### 2.2. Experimental Fish, Treatments, and Environmental Conditions

In this study, a magnetic device manufactured by Delta Water Co. (Alexandria, Egypt) was employed for water treatment. The device has a magnetic intensity of 14,500 Gauss (1.45 Tesla) and is constructed with an inner magnet enclosed in a copper housing, along with an outer magnet shielded by a steel casing. It is designed with unidirectional inlet and outlet channels to control the flow of water through the magnetic field. As water passed through the magnetic field, it became magnetized, leading to physical alterations in the composition and structure of water molecules. To verify magnetization, a handheld magnetometer was used to measure the magnetic field strength at the outlet point of the device. The study used Nile tilapia fingerlings that were obtained from a commercial hatchery in Kafr El-Shikh Governorate, Egypt. The fish were kept for two weeks to adjust to their new environment before starting the experiment. Using a 3 × 2 factorial design, three levels of dietary crude protein (25%, 30%, and 35%) and two water types (magnetized and non-magnetized) were tested for 10 weeks. A total of 180 fish with an average initial weight of 4.13 ± 0.004 g were divided randomly into six different treatments. Each group had three fiberglass tanks containing about 60 L of water, with ten fish placed in each tank.The feeding schedule and times were the same as during the two-week adjustment phase. The tanks were kept on a 12 h light/12 h dark cycle for the whole experiment. Every four days, 50% of the water volume in each tank was drained out and replaced with magnetized water to remove wastes. Air pumps continuously circulated oxygen in the water. The fish were hand-fed three times a day at 8 am, 12 pm, and 4 pm. They received an amount equal to 3% of their body weight until they appeared to be satiated. The amount of feed given was adjusted every two weeks based on new weight measurements over the 70-day trial period.

### 2.3. Water Quality Parameters

Physicochemical parameters were assessed fortnightly by collecting water samples from each aquarium at a depth of 20 cm. On-site, a digital pH meter (Fisher Scientific, Waltham, MA, USA) was used to measure the pH. Daily measurements of dissolved oxygen (DO) and water temperature at 8 am were taken using an oxygen and temperature meter manufactured by Jenway (London, UK), following the APHA [21] guidelines. To estimate the concentration of unionized ammonia (NH_3_) in the water samples, a multi-parameter analyzer (HANNA Instruments, Smithfield, RI, USA) was used, following the method described by Boyd [22]. Nitrite–nitrogen concentration was measured using the diazotization method with the same spectrophotometer at a wavelength of 543 nm. Monitoring these physicochemical parameters on a regular basis allowed for the assessment of water quality in the aquariums. By employing specific instruments and methods referenced from APHA [21] and Boyd [22], the concentrations of pH, dissolved oxygen, water temperature, unionized ammonia, nitrate, and nitrite–nitrogen could be accurately determined.

### 2.4. Growth and Feed Utilization

At the start of the experiment, the initial average body weight of the fish was recorded. Subsequently, the weight of all fish in each fiberglass tank was measured biweekly throughout the study period. Using the collected body weight data, various parameters were calculated as follows:

Average daily gain (ADG) = (wt_2_ − wt_1_)/t

Specific growth rate (SGR%/day) = [(ln W_2_ − ln W_1_) × 100]/t

where *W*_1_ and *W*_2_ represent the initial and final body weights (g), respectively, and *t* is the duration of the experimental period in days.

Feed conversion ratio (FCR) = Total feed fed (g)/Body weight gain (g)

Survival rate = (Number of surviving fish/Initial number of fish) × 100

### 2.5. Blood Serum Biochemical, Immunity, and Antioxidant Parameters

At the end of the experimental period, the fish were fasted for 24 h to clear their digestive tracts. Following this, three fish from each tank were anesthetized with MS-222 at a dose of 100 mg/L to facilitate blood sampling from the caudal vein. Blood was drawn using syringes without anticoagulant and then centrifuged at 3000× *g* for 15 min at 4 °C to separate the serum. The resulting serum was stored at −20 °C until further biochemical analysis. Antioxidant enzymes—including glutathione peroxidase (GPx), catalase (CAT), and superoxide dismutase (SOD)—as well as malondialdehyde (MDA), an indicator of lipid peroxidation, were quantified using commercially available assay kits (BioSource Inc., San Diego, CA, USA). Lysozyme (LZM) activity was assessed following the methodology described by Ghareghanipoora et al. [23]. Serum biochemical parameters, such as triglycerides, glucose, total protein (TP), albumin (ALB), creatinine, and uric acid, were measured using colorimetric diagnostic kits obtained from Bio-Diagnostics (Cairo, Egypt). Globulin (GLO) levels were derived by subtracting albumin from total protein, and the albumin-to-globulin ratio (AGR) was calculated using the formula: AGR = albumin/(total protein − albumin). Activities of liver enzymes, including alanine aminotransferase (ALT) and aspartate aminotransferase (AST), were measured using colorimetric kits obtained from Bio-Diagnostics. Alkaline phosphatase (ALP) activity was assessed based on the procedure described by Tietz [24]. For digestive enzyme analysis, serum lipase and amylase activities were determined using diagnostic kits provided by Biodiagnostic Co. (Giza, Egypt), following the manufacturer’s protocols, as outlined by Shihabi and Bishop [25] and Bernfeld [26], respectively. Serum protease activity was determined using a commercial colorimetric kit (Biodiagnostic Co., Egypt), with results calibrated against a bovine serum albumin standard curve.

### 2.6. Statistical Analysis

The experimental data were first assessed for normality using the Shapiro–Wilk test. Once the assumption of normal distribution was verified, a two-way analysis of variance (ANOVA) was conducted to evaluate the effects of the studied factors and their interaction. Differences were deemed statistically significant at a probability level of *p* < 0.05. In cases where significant effects were detected, Tukey’s multiple-comparison test was applied to determine specific differences between treatment groups. All statistical analyses were performed using SAS software (Version 9.00) [27].

## 3. Results

### 3.1. Water Quality

Table 2 illustrates the effects of water magnetic treatment and dietary crude protein levels on water quality parameters in *O. niloticus* culture. Magnetic treatment significantly increased dissolved oxygen and pH levels compared to untreated water. Additionally, it significantly reduced concentrations of ammonia, nitrite, and nitrate. However, it had no effect on water temperature. Dietary protein level had no statistically significant influence on water temperature, dissolved oxygen, ammonia, nitrite, or nitrate concentrations. Although pH showed a trend toward higher values at lower protein levels, the differences were not statistically significant (*p* = 0.092). No significant interaction was observed between magnetic treatment and dietary crude protein level for any of the assessed water quality parameters.

### 3.2. Growth Performance

As shown in Table 3, the growth performance and feed efficiency parameters of Nile tilapia were significantly affected by water magnetic treatment and dietary protein levels. Water magnetic treatment significantly improved the final weight (*p* = 0.005), ADG (*p* = 0.004), SGR (*p* = 0.004), and FCR (*p* < 0.001), but did not significantly affect feed intake (*p* = 0.107) or survival rate (*p* = 0.069). Increasing the protein level from 25% to 35% led to a significant increase in final weight (*p* < 0.001), ADG (*p* < 0.001), and SGR (*p* < 0.001) and a significant improvement in FCR (*p* < 0.001). Feed intake decreased significantly with increasing dietary protein (*p* = 0.011), and survival rate improved significantly in the 30%- and 35%-protein groups compared to the 25% group (*p* = 0.047). A significant interaction between water type and dietary protein level was observed only for survival rate (*p* = 0.047). The lowest survival rate was recorded in fish fed the 25%-protein diet without magnetic treatment, whereas all other groups exhibited a 100% survival rate.

### 3.3. Carcass Composition

Water magnetic treatment significantly increased crude protein content in fish (*p* = 0.016) but had no significant effect on moisture, crude lipids, or ash (Table 4). Dietary protein levels significantly affected all body composition parameters except ash. Moisture content decreased with increasing dietary protein levels (*p* = 0.005). Crude protein increased significantly, reaching the highest values with the 35%-protein diet (*p* < 0.001). Crude lipids were highest at 25% protein and declined as protein levels increased (*p* < 0.001). A significant interaction was found for ash content (*p* < 0.001). Fish fed the 35%-protein diet without magnetic treatment had the highest ash content, whereas magnetic treatment reduced ash levels to those comparable with the 25%-protein groups (Table 4).

### 3.4. Liver and Kidney Function Parameters

The AST, ALT, and ALP results revealed significant differences in their activities in relation to dietary crude protein levels and water magnetic treatment and their interaction (Table 5). Magnetic water treatment significantly reduced the activities of AST, ALT, and ALP, as well as urea concentration (*p* < 0.001). Dietary protein level had a significant effect on AST, ALT, ALP (*p* ≤ 0.001), urea (*p* = 0.001), and uric acid (*p* = 0.001). AST and ALT activity were noticeably elevated in fish consuming the 25%-protein diet compared to those on the 30%- and 35%-protein diets. The highest ALP activity was observed in the 25%- and 30%-protein groups, with a significant decline at 35%. Uric acid concentration increased significantly with increasing protein level, whereas urea showed an inverse trend. A significant interaction (*p* = 0.030) was only found for urea levels, where fish fed 25%- and 30%-protein diets without magnetic treatment had higher urea compared to those receiving magnetized water at the same protein levels (Table 5).

### 3.5. Blood Biochemical Parameters

Table 6 presents the impact of magnetized water, dietary protein levels, and their interaction on selected blood biochemical parameters of Nile tilapia. Fish groups reared in magnetized water showed significantly (*p* < 0.001) higher concentrations of TP, ALB, and GLO compared to those reared in unmagnetized water. Conversely, the use of magnetized water significantly (*p* < 0.001) reduced blood glucose, cholesterol, and triglyceride levels. No significant effect (*p* > 0.05) was observed on the ALB/GLO ratio. Increasing dietary protein from 25% to 35% led to significant (*p* < 0.05 or 0.001) increases in TP, ALB, and GLO concentrations, as well as in blood cholesterol and triglyceride levels. A significant interaction (*p* < 0.05 or < 0.001) between water type and dietary protein was found for TP, GLO, and triglyceride concentrations. The highest TP, ALB, and GLO levels were observed in fish fed 35% protein with magnetized water, while the lowest were recorded in fish fed 25% protein without magnetized water. Triglyceride levels were lowest in the group that received 25% protein with magnetized water and highest in all non-magnetized groups, regardless of protein level. No significant interactions were found for ALB, ALB/GLO ratio, glucose, or cholesterol concentrations.

### 3.6. Antioxidant Status and Immune Response

**Figure 1:**
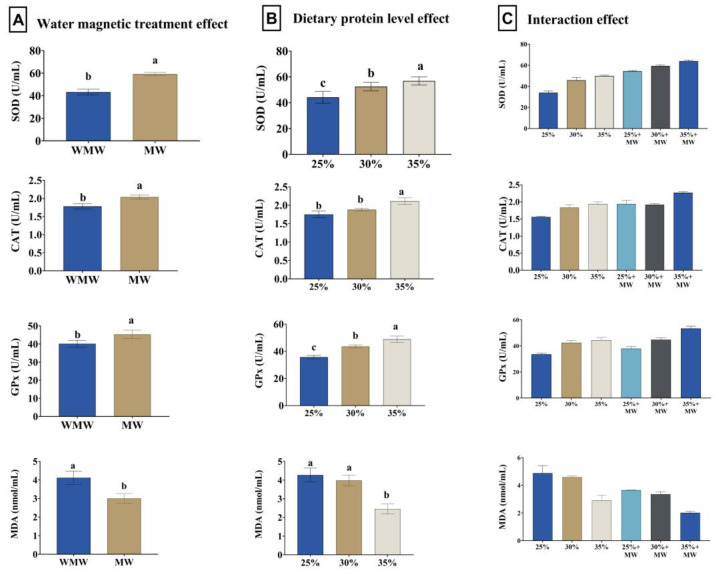
Effect of water magnetic treatment (**A**), dietary protein level (**B**), and their interaction (**C**) for 70 days on superoxide dismutase (SOD), catalase (CAT), glutathione peroxidase (GPx), and malondialdehyde (MDA) levels in the serum of Nile tilapia. Data expressed as means ± SEs. Bars carrying different letters (a, b, and c) differ significantly.

**Figure 2:**
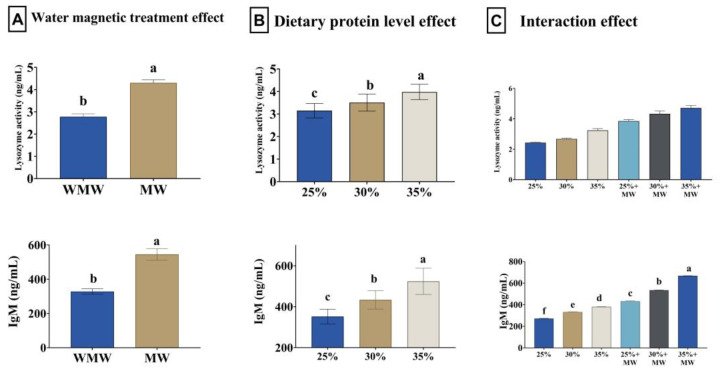
Effect of water magnetic treatment (**A**), dietary protein level (**B**), and their interaction (**C**) for 70 days on lysozyme activity and immunoglobulin M (IgM) level in the serum of Nile tilapia. Data expressed as means ± SEs. Bars carrying different letters (a–f) differ significantly.

Magnetized water significantly increased the activities of CAT, GPx, and SOD, along with a marked reduction in MDA levels (*p* = 0.002 and *p* < 0.001). Dietary protein levels also significantly affected these antioxidant parameters, with higher protein levels (30% and 35%) associated with enhanced antioxidant enzyme activities and reduced oxidative stress markers (*p* < 0.001). The interaction between magnetized water and protein level was not statistically significant for these indices (Figure 1). Both magnetized water and increased dietary protein significantly enhanced lysozyme activity and IgM levels (*p* < 0.001). A significant interaction effect was found for IgM (*p* < 0.001), with the highest levels observed in fish fed 35% protein and magnetized water (Figure 2).

### 3.7. Digestive Enzymes

**Figure 3:**
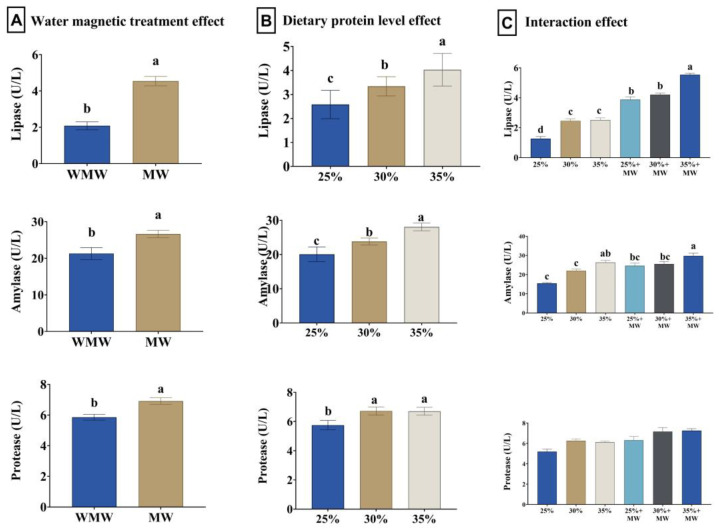
Effect of water magnetic treatment (**A**), dietary protein level (**B**), and their interaction (**C**) for 70 days on the activity of lipase, amylase, and protease in the serum of Nile tilapia. Data expressed as means ± SEs. Bars carrying different letters (a–d) differ significantly.

Magnetized water significantly enhanced the activity of lipase, amylase, and protease (*p* < 0.001). Dietary protein levels significantly increased lipase, protease, and amylase activities (*p* < 0.001 and 0.005). The interaction between water type and dietary protein was significant for lipase and amylase (*p* = 0.002 and 0.035, respectively), while no significant interaction was found for protease. The combination of magnetized water and 35% dietary protein resulted in the highest enzymatic activities (Figure 3).

## 4. Discussion

The present study demonstrated that the application of magnetic treatment to water exhibited significantly higher dissolved DO and pH, while ammonia, nitrite, and nitrate concentrations were significantly lower than in the control water. These findings align with previous studies of magnetic water treatment in aquaculture systems. Ahmed and Abd El-Hamed [9] similarly reported that magnetized water (2000 gauss) yielded higher DO and pH levels, along with lower ammonia levels, while water temperature was unaffected. Helmy et al. [12] also observed slight but significant increases in DO and pH and concurrent reductions in total ammonia in a tilapia aquaponics system with magnetized water. The rise in DO can be attributed to several factors related to the altered properties of water under magnetic influence. These changes impact water parameters, molecular structure, and ultimately oxygen solubility and transfer [28]. Indeed, increased DO in magnetized water has been noted in other experiments [12,29].

The elevation in pH observed here has also been reported by some other authors. In our study, magnetization produced a modest pH increase compared to control water. Alkhazan and Saddiq [30] likewise found higher pH with magnetic treatment, whereas El-Sayed et al. [31] reported the opposite effect (a pH decrease). Variability in pH response may depend on water chemistry and magnet strength. In general, magnetic fields can alter hydrogen bonding and ionic dissociation in water, sometimes leading to softening and increased carbonate availability (which raises pH) [32]. Our results indicated that the use of magnets in water treatment led to a reduction in unionized NH_3_ levels compared to the control group. This observation is consistent with the findings of Hassan et al. [13], who reported better management of ammonia–nitrogen using magnetized water. The observed decline in ammonia concentrations may be attributed to the oxidation of NH_3_ into NO_2_^−^ and NO_3_^−^ [9], which was supported by Ahmed and Abd El-Hamed [9] and Aziz et al. [29], who noted increased DO and pH levels in magnetized water alongside a reduction in ammonia.

In this experiment, magnetic water treatment significantly enhanced the final weight, ADG, SGR, and FCR in Nile tilapia. These improvements are consistent with previous studies reporting that magnetic treatment of water can modify its physicochemical properties, leading to better nutrient solubility and bioavailability, which in turn enhance fish metabolism and growth performance [9,19]. Additionally, Tyari et al. [33] suggested that magnetic water facilitates the transfer of nutrients throughout the body, improving overall performance. According to Brizhik [34], magnetic fields may influence biological systems through a series of cascading effects, beginning with changes in electrosoliton dynamics, leading to alterations in macromolecular states, impacting respiration rates and ultimately affecting the organism’s overall metabolic processes. Likewise, Irhayyim et al. [35] observed improved feed utilization and growth indicators in common carp by incorporating magnetized water into a recirculating aquaculture system. On the other hand, increasing dietary protein levels from 25% to 35% resulted in significant improvements in final weight, ADG, SGR, and FCR, while feed intake decreased and survival rates improved in the higher-protein groups. These findings align with established knowledge that protein is a critical nutrient for fish growth, providing essential amino acids for tissue synthesis [7,36]. The decrease in feed intake with higher protein levels may be attributed to several physiological mechanisms, primarily related to metabolic demands and appetite regulation. Research indicated that as dietary protein levels increase, the associated oxygen demand also rises, which can limit voluntary feed intake due to the fish’s metabolic capacity for oxygen use [37]. Additionally, higher-protein diets influence the expression of appetite-regulating hormones and digestive enzymes, which can further modulate feed intake [38]. The improvement in survival rates with higher-protein diets could possibly be due to the fact that adequate protein intake supports better health and resilience in fish, possibly by enhancing immune function and stress tolerance [39]. Notably, magnetic water treatment was associated with a 100% survival rate across all treatment groups. This outcome is consistent with previous findings by Hassan et al. [13], who reported high survival rates (88.9–95.8%) in Jade Perch exposed to a magnetic field, with no evidence of adverse effects.

Herein, magnetic water treatment significantly increased the crude protein content in fish, while having no significant effect on moisture, crude lipids, or ash content. These results align with previous studies suggesting that magnetic treatment of water can enhance nutrient assimilation and metabolic efficiency in fish. For instance, Hassan et al. [13] reported that magnetized water positively affected body weight gain and crude protein content in *Scortum barcoo*. Similarly, El-Sayed et al. [31] observed a higher crude protein content in sea bass larvae reared in magnetized water. In this study, increasing dietary protein levels from 25% to 35% significantly increased crude protein content, but decreased moisture and crude lipid contents. These findings are consistent with Abdel-Tawwab et al.’s [7] findings that higher dietary protein levels enhance protein deposition and reduce lipid accumulation in fish. Several studies have shown that crude protein content increased in fish bodies with increasing dietary protein [40,41,42]. The rise in deposited nutrients can be attributed to enhanced nutrient utilization and high digestibility. Variations in the protein and lipid contents of fish bodies may be associated with shifts in their synthesis, muscle deposition rates, and/or differences in growth rates [43].

Magnetic water treatment notably reduced the activities of AST, ALT, and ALP, along with urea concentrations. This hepatoprotective effect is possibly due to enhanced antioxidant capacity and improved cellular metabolism [44]. Similar outcomes were observed in studies where magnetized water administration led to decreased liver enzyme activities and improved oxidative status in animal models [45,46]. Lower-protein diets can induce metabolic stress, leading to increased liver enzyme activities. The 25%-protein diet resulted in higher ALT and AST levels, indicating potential liver stress or damage due to inadequate protein for metabolic needs [47]. The hepatic MDA content was also higher in lower protein groups, suggesting increased oxidative stress, which correlates with elevated liver enzyme levels [48]. Elevated uric acid levels observed with a 35% dietary protein intake are likely attributable to increased purine metabolism. High-protein diets, particularly those rich in animal-based proteins, provide substantial amounts of purines [49]. The liver metabolizes purines into uric acid, which is then excreted [50]. Several studies have linked high-protein diets to increased serum uric acid levels in various organisms, including fish [51].

Treatment of Nile tilapia with magnetized water resulted in increased levels of TP, ALB, and GLO in the blood, while significantly reducing cholesterol, glucose, and triglyceride concentrations. The improvement in protein profiles may be associated with enhanced protein metabolism [52]. Magnetized water may induce metabolic mechanisms that enhance protein synthesis, resulting in elevated levels of TP and particular proteins like ALB and GLO [29]. Additionally, studies suggest that magnetized water can reduce blood cholesterol and triglyceride levels, likely due to enhanced lipid metabolism and decreased absorption of dietary fats [53]. The observed reduction in blood glucose may also be attributed to improved metabolic efficiency and increased insulin sensitivity in fish exposed to magnetized water [29]. Increasing dietary protein from 25% to 35% in Nile tilapia diets led to elevated blood TP, ALB, and GLO levels, likely due to enhanced amino acid availability, which supports protein synthesis and immune function [5]. The increase in blood cholesterol may be attributed to increased intake of protein-associated lipids or enhanced hepatic lipid metabolism, as higher protein levels can stimulate liver activity involved in both protein and lipid processing [54].

Magnetized water has the potential to modify the physicochemical characteristics of water molecules, including lowering surface tension and mitigating the damaging effects of free radicals by slowing down the chemical reactions that harm lipids, proteins, and DNA. Magnetized water has been shown to positively influence the antioxidant capacity and immune response while reducing oxidative stress [55]. Increasing dietary protein from 25% to 35% in Nile tilapia diets enhanced antioxidant enzymes (SOD, GPx, and CAT) and immunity markers (lysozyme activity and IgM levels), likely due to the greater availability of essential amino acids that support the synthesis of antioxidant proteins and immune-related molecules [56]. Higher protein intake improves the fish’s overall metabolic capacity, enabling a more effective defense against oxidative stress through elevated production of endogenous antioxidants [57]. Additionally, amino acids are known to modulate immune responses, contributing to increased lysozyme activity and IgM production [56].

The enhancement of digestive enzyme activity in Nile tilapia through magnetized water treatment could be due to the multifaceted process involving improved water quality, stimulation of digestive glands, modulation of gut health, and overall metabolic benefits [29,58,59]. This finding is in agreement with the results reported by Tang et al. [19]. Increasing dietary protein from 25% to 35% in Nile tilapia diets led to elevated activities of digestive enzymes, protease, lipase, and amylase, due to enhanced stimulation of the digestive system in response to greater nutrient intake. Higher protein levels increase the demand for digestion and absorption, particularly of proteins and associated macronutrients, which in turn promotes the upregulation of enzyme secretion by the pancreas and the intestinal mucosa [60,61]. Specifically, protease activity increases to break down the greater protein load, while lipase and amylase are also stimulated to support efficient digestion of dietary fats and carbohydrates that accompany high-protein feeds.

## 5. Conclusions

The overall results indicate that magnetic water treatment, across all protein levels, significantly enhanced growth performance, FCR, survival, blood biochemical parameters, immunity, antioxidant status, and digestive enzyme activities. The best interactive outcomes were observed in fish fed a 35%-protein diet with magnetic water, as evidenced by reduced serum urea levels and enhanced concentrations of total protein, globulin, triglycerides, and IgM, along with increased activities of lipase and amylase enzymes. Lower protein or the absence of magnetic treatment resulted in reduced performance in these key indicators. Future research should investigate the molecular mechanisms underlying the effects of magnetic water treatment to better understand how it enhances specific biological functions. Moreover, assessing its effectiveness in commercial-scale systems and across different fish species could support wider adoption in aquaculture practices.

## Figures and Tables

**Table 1:** Ingredients and proximate composition of the experimental diets (g/100 g) on a dry matter basis.

	Crude Protein Levels		
	25%	30%	35%
Ingredients			
Fish meal 65%	10	10	10
Soybean meal 46%	25	37	50
Yellow corn	51	39	26
Rice bran	7	7	7
Corn gluten	4	4	4
Fish oil	1	1	1
Vegetable oil	1	1	1
Premix ^1^	0.5	0.5	0.5
Salt	0.5	0.5	0.5
Proximate composition			
Crude protein (N × 6.25)	25.46	30.06	35.04
Crude lipids	6.49	6.51	6.54
Crude fiber	2.23	2.16	2.07
Ash	6.89	7.28	7.51

^1^ Provides the following per kg of diet: vitamin D3: 600 IU, vitamin A: 4000 IU, vitamin E: 20 mg, vitamin B1: 3.6 mg, vitamin K3: 5 mg, vitamin B2: 6 mg, vitamin B3: 14.4 mg, vitamin B5: 12 mg, vitamin B12: 0.02 mg, vitamin B6: 3.5 mg, folic acid: 0.9 mg, biotin: 0.07 mg, vitamin C: 50 mg, inositol: 300 mg, Fe: 30 mg, Mg: 15 mg, Cu: 4 mg, Zn: 42 mg, Co: 0.11 mg, K: 75 mg, Se: 0.04 mg, Mn: 1.6 mg, I: 0.4 mg, and Mo: 0.005 mg.

**Table 2:** Effect of water magnetic treatment and dietary protein levels on water quality parameters in Nile tilapia (*Oreochromis niloticus*) culture.

Magnetic Treatment	Dietary Crude Protein	Temperature (°C)	Dissolved Oxygen (mg/L)	pH	Ammonia (NH_3_, mg/L)	Nitrite (NO_2_^−^, mg/L)	Nitrate (NO_3_^−^, mg/L)
Individual treatment means							
Without	25%	28.67 ± 0.63	5.61 ± 0.36	7.16 ± 0.10	0.17 ± 0.03	0.08 ± 0.01	0.007 ± 0.001
	30%	28.71 ± 0.67	5.67 ± 0.26	7.07 ± 0.05	0.18 ± 0.03	0.08 ± 0.01	0.007 ± 0.001
	35%	28.73 ± 0.63	5.65 ± 0.19	6.99 ± 0.09	0.17 ± 0.04	0.10 ± 0.01	0.008 ± 0.001
With	25%	28.88 ± 0.49	8.61 ± 0.46	7.60 ± 0.09	0.08 ± 0.01	0.02 ± 0.01	0.001 ± 0.0003
	30%	28.74 ± 0.54	8.91 ± 0.50	7.49 ± 0.09	0.08 ± 0.01	0.04 ± 0.01	0.002 ± 0.001
	35%	28.56 ± 0.62	8.98 ± 0.53	7.39 ± 0.08	0.09 ± 0.01	0.05 ± 0.02	0.003 ± 0.001
Water magnetic treatment effect							
Without		28.70 ± 0.35	5.64 ± 0.15	7.07 ± 0.05	0.17 ± 0.02	0.09 ± 0.01	0.007 ± 0.001
With		28.73 ± 0.30	8.83 ± 0.27	7.49 ± 0.05	0.08 ± 0.01	0.03 ± 0.01	0.002 ± 0.0003
Dietary crude protein effect							
	25%	28.78 ± 0.38	7.11 ± 0.53	7.38 ± 0.09	0.12 ± 0.02	0.05 ± 0.01	0.004 ± 0.001
	30%	28.73 ± 0.41	7.29 ± 0.56	7.28 ± 0.08	0.13 ± 0.02	0.06 ± 0.01	0.005 ± 0.001
	35%	28.64 ± 0.42	7.32 ± 0.57	7.19 ± 0.08	0.13 ± 0.02	0.07 ± 0.01	0.005 ± 0.001
Two-way ANOVA: *p*-values							
Interaction		0.950	0.912	0.987	0.867	0.788	0.948
Water magnetic treatment		0.955	˂0.001	˂0.001	˂0.001	˂0.001	˂0.001
Dietary crude protein		0.975	0.854	0.092	0.968	0.270	0.134

Means ± SEs are presented for each parameter. Without = non-magnetized water; With = magnetized water.

**Table 3:** Growth performance and feed efficiency parameters of Nile tilapia (*Oreochromis niloticus*) under water magnetic treatment and dietary crude protein levels.

Magnetic Treatment	Dietary Crude Protein	IW (g/fish)	FW (g/fish)	ADG (g/day)	Feed Intake (g/fish)	SGR (%/day)	FCR	SR (%)
Individual treatment means								
Without	25%	4.13 ± 0.001	14.65 ± 0.45	0.150 ± 0.006	21.79 ± 0.55	1.81 ± 0.04	2.07 ± 0.04	93.33 ± 3.33 ^b^
	30%	4.15 ± 0.01	15.87 ± 0.43	0.167 ± 0.006	19.80 ± 0.89	1.92 ± 0.04	1.69 ± 0.02	100 ± 0.00 ^a^
	35%	4.13 ± 0.02	17.25 ± 0.36	0.187 ± 0.005	21.62 ± 0.38	2.04 ± 0.03	1.65 ± 0.04	100 ± 0.00 ^a^
With	25%	4.12 ± 0.01	15.10 ± 0.37	0.157 ± 0.005	21.62 ± 0.26	1.86 ± 0.04	1.97 ± 0.05	100 ± 0.00 ^a^
	30%	4.12 ± 0.01	17.66 ± 0.16	0.193 ± 0.002	19.66 ± 0.32	2.08 ± 0.01	1.45 ± 0.04	100 ± 0.00 ^a^
	35%	4.13 ± 0.002	18.10 ± 0.34	0.200 ± 0.005	19.63 ± 0.57	2.11 ± 0.03	1.41 ± 0.08	100 ± 0.00 ^a^
Water magnetic treatment effect								
Without		4.14 ± 0.01	15.92 ± 0.43	0.168 ± 0.006	22.18 ± 0.45	21.07 ± 0.04	1.80 ± 0.07	97.78 ± 1.47
With		4.12 ± 0.004	16.96 ± 0.49	0.183 ± 0.007	18.97 ± 0.39	20.30 ± 0.04	1.61 ± 0.09	100 ± 0.00
Dietary protein level effect								
	25%	4.12 ± 0.01	14.88 ± 0.28 ^c^	0.154 ± 0.004 ^c^	21.71 ± 0.27 ^a^	1.83 ± 0.03 ^c^	2.02 ± 0.04 ^a^	96.67 ± 2.11 ^b^
	30%	4.14 ± 0.01	16.77 ± 0.45 ^b^	0.180 ± 0.006 ^b^	19.73 ± 0.42 ^b^	2.00 ± 0.04 ^b^	1.57 ± 0.06 ^b^	100 ± 0.00 ^a^
	35%	4.13 ± 0.01	17.68 ± 0.29 ^a^	0.193 ± 0.004 ^a^	20.62 ± 0.54 ^ab^	2.08 ± 0.02 ^a^	1.53 ± 0.07 ^b^	100 ± 0.00 ^a^
Two-way ANOVA: *p*-values								
Interaction		0.233	0.210	0.198	0.185	0.204	0.287	0.047
Water magnetic treatment		0.098	0.005	0.004	0.107	0.004	˂0.001	0.069
Dietary protein level		0.466	˂0.001	˂0.001	0.011	˂0.001	˂0.001	0.047

Means in the same column with different superscripts are significantly different (*p* ˂ 0.05). Treatment means represent the average values of three tanks per treatment. Means ± SEs are presented for each parameter. Without = non-magnetized water; With = magnetized water. IW: Initial weight; FW: Final weight; ADG: Average daily gain; SGR: Specific growth rate; FCR: Feed conversion ratio; SR: Survival rate.

**Table 4:** Effects of water magnetic treatment and dietary crude protein levels on the whole-body composition (% on a wet basis).

Magnetic Treatment	Dietary Crude Protein	Moisture	Crude Lipids	Ash	Crude Protein
Individual treatment means					
Without	25%	77.11 ± 0.57	4.00 ± 0.14	4.10 ± 0.14 ^b^	13.91 ± 0.28
	30%	76.35 ± 0.37	3.30 ± 0.04	4.31 ± 0.09 ^b^	15.06 ± 0.28
	35%	74.55 ± 0.32	3.33 ± 0.03	4.73 ± 0.08 ^a^	16.41 ± 0.28
With	25%	76.14 ± 0.54	4.00 ± 0.04	4.79 ± 0.09 ^a^	14.51 ± 0.35
	30%	75.12 ± 0.25	3.20 ± 0.04	4.71 ± 0.06 ^a^	16.01 ± 0.08
	35%	74.96 ± 0.54	3.20 ± 0.04	4.19 ± 0.14 ^b^	16.75 ± 0.30
Water magnetic treatment effect					
Without		76.00 ± 0.41	3.54 ± 0.12	4.38 ± 0.11	15.13 ± 0.39
With		75.41 ± 0.25	3.47 ± 0.14	4.56 ± 0.11	15.76 ± 0.36
Dietary crude protein effect					
	25%	76.63 ± 0.41 ^a^	4.00 ± 0.07 ^a^	4.45 ± 0.17	14.21 ± 0.24 ^c^
	30%	75.12 ± 0.25 ^ab^	3.25 ± 0.03 ^b^	4.51 ± 0.10	15.54 ± 0.25 ^b^
	35%	74.76 ± 0.30 ^b^	3.27 ± 0.04 ^b^	4.46 ± 0.14	16.58 ± 0.20 ^a^
Two-way ANOVA: *p*-values					
Interaction		0.190	0.581	˂0.001	0.558
Water magnetic treatment		0.129	0.190	0.058	0.016
Dietary crude protein		0.005	˂0.001	0.834	˂0.001

Means ± SEs are presented for each parameter. Means in the same column with different superscripts are significantly different (*p* ˂ 0.05). Without = non-magnetized water; With = magnetized water.

**Table 5:** Effect of using magnetized water on liver and kidney function parameters of blood of Nile tilapia fed on 25, 30, and 35% CP diets.

Magnetic Treatment	Dietary Crude Protein	AST (IU/L)	ALT (IU/L)	ALP (IU/L)	Creat. (mg/dL)	UA (mg/dL)	Urea (mg/dL)
Individual treatment means							
Without	25%	43.46 ± 1.87	25.79 ± 0.70	39.11 ± 0.73	0.21 ± 0.02	1.62 ± 0.11	6.27 ^a^ ± 0.19
	30%	36.89 ± 1.37	22.33 ± 0.92	39.49 ± 1.45	0.16 ± 0.02	1.73 ± 0.08	5.84 ^a^ ± 0.38
	35%	37.02 ± 0.41	22.72 ± 0.40	34.68 ± 0.46	0.14 ± 0.02	1.99 ± 0.11	4.67 ^b^ ± 0.09
With	25%	37.76 ± 0.25	19.71 ± 0.37	29.50 ± 0.65	0.13 ± 0.02	1.52 ± 0.10	4.24 ^bc^ ± 0.14
	30%	32.59 ± 0.54	16.38 ± 0.56	28.53 ± 0.54	0.17 ± 0.03	1.63 ± 0.05	3.94 ^c^ ± 0.13
	35%	31.74 ± 0.43	15.86 ± 0.43	26.53 ± 0.58	0.11 ± 0.02	1.96 ± 0.06	3.80 ^c^ ± 0.17
Water magnetic treatment effect							
Without		39.12 ± 1.28	23.61 ± 0.65	37.76 ± 0.91	0.17 ± 0.01	1.78 ± 0.07	5.59 ± 0.27
With		34.03 ± 0.96	17.32 ± 0.65	28.19 ± 0.53	0.14 ± 0.01	1.70 ± 0.08	3.99 ± 0.10
Dietary crude protein effect							
25%		40.61 ^a^ ± 1.53	22.75 ^a^ ± 1.40	34.31 ^a^ ± 2.19	0.17 ± 0.02	1.57 ^b^ ± 0.07	5.26 ^a^ ± 0.47
30%		34.74 ^b^ ± 1.17	19.36 ^b^ ± 1.42	34.01 ^a^ ± 2.55	0.17 ± 0.02	1.68 ^b^ ± 0.03	4.89 ^a^ ± 0.46
35%		34.38 ^b^ ± 1.21	19.29 ^b^ ± 1.52	30.61 ^b^ ± 1.85	0.13 ± 0.01	1.98 ^a^ ± 0.06	4.24 ^b^ ± 0.21
Two-way ANOVA: *p*-values							
Interaction		0.779	0.718	0.257	0.132	0.889	0.030
Water magnetic treatment		<0.001	<0.001	<0.001	0.070	0.280	<0.001
Dietary crude protein		<0.001	<0.001	0.001	0.095	0.001	0.001

Means in the same column with the same superscript letters are not significantly different (*p* < 0.05). Without = non-magnetized water; With = magnetized water.

**Table 6:** Effect of using magnetized water on some biochemical parameters of blood of Nile tilapia fed on 25, 30, and 35% CP diets.

Magnetic Treatment	Dietary Crude Protein	TP (g/dL)	ALB (g/dL)	GLO (g/dL)	ALB/GLO	Glucose mg/dL	Cholesterol mg/dL	Triglycerides mg/dL
Individual treatment means								
Without	25%	1.65 ^c^ ± 0.04	0.82 ± 0.05	0.83 ^d^ ± 0.02	0.99 ± 0.08	72.27 ± 0.54	144.50 ± 0.71	168.23 ^a^ ± 1.45
	30%	1.74 ^c^ ± 0.05	0.81 ± 0.05	0.90 ^d^ ± 0.03	0.90 ± 0.09	78.69 ± 0.49	146.33 ± 2.11	170.57 ^a^ ± 0.38
	35%	2.33 ^b^ ± 0.08	1.11 ± 0.10	1.22 ^c^ ± 0.03	0.91 ± 0.09	76.23 ± 0.71	160.18 ± 3.92	169.23 ^a^ ± 1.42
With	25%	2.48 ^b^ ± 0.09	1.21 ± 0.10	1.27 ^c^ ± 0.01	0.95 ± 0.08	62.25 ± 1.75	130.75 ± 1.44	139.72 ^d^ ± 1.41
	30%	2.94 ^a^ ± 0.10	1.35 ± 0.07	1.56 ^b^ ± 0.04	0.86 ± 0.03	64.84 ± 1.23	140.63 ± 2.03	145.63 ^c^ ± 1.41
	35%	3.12 ^a^ ± 0.08	1.46 ± 0.03	1.66 ^a^ ± 0.05	0.88 ± 0.02	65.94 ± 1.01	155.25 ± 0.64	159.25 ^b^ ± 1.42
Water magnetic treatment effect								
Without		1.91 ± 0.11	0.91 ± 0.06	0.98 ± 0.06	0.94 ± 0.05	75.73 ± 0.98	150.34 ± 2.80	169.35 ± 0.69
With		2.85 ± 0.11	1.34 ± 0.05	1.49 ± 0.06	0.90 ± 0.03	64.34 ± 0.88	142.21 ± 3.63	148.20 ± 2.98
Dietary crude protein effect								
25%		2.07 ^c^ ± 0.19	1.02 ± 0.10	1.05 ^c^ ± 0.10	0.97 ± 0.05	67.26 ± 2.39	137.63 ± 3.16	153.98 ^c^ ± 6.44
30%		2.34 ^b^ ± 0.27	1.08 ± 0.13	1.23 ^b^ ± 0.15	0.88 ± 0.04	71.77 ± 3.15	143.48 ± 1.83	158.10 ^b^ ± 5.61
35%		2.73 ^a^ ± 0.18	1.29 ± 0.09	1.44 ^a^ ± 0.10	0.88 ± 0.02	71.09 ± 2.37	157.72 ± 2.09	164.24 ^a^ ± 2.41
Two-way ANOVA: *p*-values								
Interaction		0.033	0.386	0.006	0.995	0.169	0.111	<0.001
Water magnetic treatment		<0.001	<0.001	<0.001	0.526	<0.001	0.001	<0.001
Dietary crude protein		<0.001	0.006	<0.001	0.426	0.002	<0.001	<0.001

Means in the same column with the same superscript letters are not significantly different (*p* < 0.05). Without = non-magnetized water; With = magnetized water.

## Data Availability

The datasets used in with this research are available from the corresponding author upon reasonable request.
